# Patient-Reported Experiences with a Low-Carbohydrate Ketogenic Diet: An International Survey in Patients with McArdle Disease

**DOI:** 10.3390/nu15040843

**Published:** 2023-02-07

**Authors:** Nicoline Løkken, Nicol C. Voermans, Linda K. Andersen, Walaa Karazi, Stacey L. Reason, Heidi Zweers, Gustav Wilms, Alfredo Santalla, Edward Susanibar, Alejandro Lucia, John Vissing

**Affiliations:** 1 Copenhagen Neuromuscular Center, Department of Neurology, Copenhagen University Hospital, Rigshospitalet, DK-2100 Copenhagen, Denmark; 2 Department of Clinical Medicine, University of Copenhagen, DK-2200 Copenhagen, Denmark; 3 The Department of Neurology, Donders Institute for Brain, Cognition and Behaviour, Radboud University Nijmegen Medical Centre, 6525 GA Nijmegen, The Netherlands; 4 International Association for Muscle Glycogen Storage Disease, Torrance, CA 90505, USA; 5 Department of Gastroenterology and Hepatology-Dietetics, Radboudumc, 6500 HB Nijmegen, The Netherlands; 6 Department of Sport and Computer Science, Section of Physical Education and Sports, Faculty of Sport, Universidad Pablo de Olavide, 41013 Seville, Spain; 7 Faculty of Sport Sciences, Universidad Europea de Madrid, 28670 Madrid, Spain; 8 Physical Activity Health Research Group (PaHerg), Research Institute of Hospital 12 de Octubre (‘i+12’), 28040 Madrid, Spain

**Keywords:** McArdle disease, ketogenic diet, low carbohydrate ketogenic diet, glycogen storage disease type V, survey, patient-reported experiences

## Abstract

The low-carbohydrate ketogenic diet (LCKD) has attracted increased attention in recent years as a potential treatment option for individuals with McArdle disease (glycogen storage disease type V), and despite the absence of strong scientific evidence of the LCKD’s benefits, increased numbers of individuals with McArdle disease have tried a LCKD. The objective of this study was to collect patient-reported experiences with a LCKD. We aimed to estimate the immediate prevalence of individuals that had tried a LCKD in an international McArdle disease cohort, and we aimed to report on the patient-reported experiences with the diet, both positive and negative. A total of 183 responses were collected from individuals with McArdle disease from 18 countries. We found that one-third of the cohort had tried a LCKD, and almost 90% experienced some degree of positive effect, with the most prominent effects on McArdle disease-related core symptoms (e.g., activity intolerance, muscle pain, and muscle fatigue). Adverse effects were rare and generally rated as mild to moderate. These patient-reported findings underline the need for randomized clinical trials to decisively determine if a LCKD is a suitable nutritional strategy for patients with McArdle disease. The results from this study can prompt and contribute to the design of such a clinical trial.

## 1. Introduction

The low-carbohydrate ketogenic diet (LCKD) has attracted increased attention in recent years as a potential treatment option for individuals with McArdle disease (glycogen storage disease type V). Yet, only two case reports [1,2] and a pilot study [3] have been published on this topic, all indicating beneficial effects of the diet. Despite the absence of strong scientific evidence of the diet’s benefits from randomized trials, increasing numbers of patients with McArdle disease have adopted a LCKD and anecdotally report positive effects on disease-specific symptoms. There are several variations of the LCKD, and the less strict variants are mostly used by adults with McArdle disease [4,5].

The patient advocacy organization (IamGSD) has, in response to the increasing interest in the LCKD, launched several initiatives [6], including establishing a social media group (‘Ketosis in McArdle’) with currently >1000 members, publishing a situation report outlining anecdotal effects of the LCKD [4]; and sponsoring a workshop (‘Future of Nutrition in McArdle disease 2018’) to connect researchers and patient advocates. Obviously, there is a patient-led demand to investigate the potential of LCKDs in McArdle disease. Theoretically, this nutritional approach may have a treatment potential based on a scientific rationale, which was also recognized in the recently published international guidelines for managing McArdle disease [7]. Individuals with McArdle disease have blocked glycogenolysis in skeletal muscle tissue due to a lack of the enzyme myophosphorylase, leading to impaired muscle energy metabolism [7,8]. Individuals experience physical activity (PA) intolerance and PA-induced muscle fatigue and pain, which can progress to contractures and rhabdomyolysis if the activity is continued despite symptoms. If, however, the intensity is reduced or the activity is paused, symptoms will subside. This stop-and-start pattern may need to be repeated a few times. After 8–10 min, PA is more easily tolerated, allowing for the resumption of PA. This phenomenon is called the “second wind” phenomenon and is due to the increased availability of alternative bloodborne fuels for muscle metabolism (i.e., free fatty acids (FFA) and hepatic-derived glucose) [7]. Ketone bodies (KB) (acetoacetate, acetone, and beta-hydroxybutyrate) are synthesized in the liver from FFA to replace blood glucose as the main energy substrate for extrahepatic tissues during periods of restricted carbohydrate availability (e.g., LCKD) [9,10,11]. In theory, a LCKD would provide alternative substrates in the form of KBs for immediate use to address the energy crisis before the “second wind”, along with a boost of both KB and FFA oxidation after the “second wind” has set in.

Anecdotal patient-reported effects of a LCKD have, among others, included improvement of PA tolerance and less muscle pain [4]. However, patient-reported experiences have not yet been systematically collected. This study, therefore, aimed to collect patient-reported experiences with LCKDs using a web-based survey amongst an international cohort of patients with McArdle disease. We aimed to estimate the prevalence of patients that had tried a LCKD and outline the patient-reported effects and overall experiences with a LCKD. The collection of patient-reported experiences is an important first step and can play a vital role in the design of future clinical trials that aim to determine whether a LCKD is a suitable nutritional strategy for the management of McArdle disease.

## 2. Materials and Methods

An international cross-sectional web-based survey was performed in 2021–2022, consisting of six parts. This study presents results from the first three parts (1–3). The remaining parts (4–6) focused on fatigue, insomnia, and quality of life and will be presented in a subsequent report. The survey was approved by the Knowledge Center for Data reviews, Capital Region, Denmark (P-2019-517) and listed on clinical trials.gov (NCT04694547). Danish regulations state that the study is exempt from reporting to the Danish Ethics Committee as there is no intervention and data are anonymized.

### 2.1. Survey Part 1

The first part focused on demographics and McArdle disease-specific symptoms. Respondents reported age, sex, height, weight, country of residence, work and family status, comorbidities, and current medications. Questions about McArdle disease included: symptom severity and frequency. The only previously proposed clinical grading scale for McArdle disease [12] was not suitable for the present study; therefore, we used a descriptive scale that we developed for this survey. Respondents graded five McArdle core symptoms (muscle pain at rest and during activity, muscle cramps/contractures, PA intolerance, and muscle fatigue) on a 5-point scale (no symptom = 1, very mild = 2, mild = 3, moderate = 4, severe = 5), resulting in a summarized McArdle symptom score ranging from a minimum of 5 to a maximum of 25 points. The respondents were instructed to grade their symptoms, as, on average, they were in a period where they were not on a LCKD. For the full survey part 1 outline, see File S1 of the supplements.

### 2.2. Survey Part 2

The second part focused on questions about the LCKD, which were designed by members of the study group (N.L., N.C.V., H.Z., J.V.) in five steps. The survey content was inspired by the output from the workshop ‘Future of Nutrition in McArdle disease 2018’. In the first step of the design process, H.Z. and N.C.V. wrote an initial survey outline, which in step two was reviewed and further developed by J.V. and N.L. Next, a patient representative (S.L.R.) reviewed the survey outline to ensure patient understanding. Fourthly, the survey part was tested among ten healthy adults to make sure all questions were clearly formulated. The last step was a final review by N.L., N.C.V., H.Z., and J.V. Questions included: ‘have you heard about the ketogenic diet?’, ‘ if yes, where have you heard of the ketogenic diet?’; ‘have you tried the ketogenic diet before (or a variation of the ketogenic diet)?’; and reasons to start a LCKD or not. A stop question ensured that only patients who had tried a LCKD were able to respond to the remaining questions in this survey part. Questions on diet experiences included LCKD variations tried, diet duration, and questions on effects (both positive and negative) from adhering to a LCKD, among other questions. As an example, patients graded the overall and specific LCKD effects on five predetermined symptoms/domains on a six-point scale from negative to excellent effect. Additionally, the patients graded the frequency of McArdle core symptoms under two different conditions. The first was to recall a period where they were not on a LCKD, and the second condition was a period where they were on the LCKD. We aimed to collect patient quotes to illustrate different experiences with a LCKD. Therefore, the last step of this survey part was a comment box, where patients could write overall comments/experiences with the LCKD. For the full survey part 2 outline, see File S2 of the supplements.

### 2.3. Survey Part 3

The third part of the survey assessed PA with the International Physical Activity Questionnaire short form (IPAQ-SF) [13,14]. The IPAQ-SF assesses PA during the last seven days at four intensity levels: (1) vigorous-intensity PA, (2) moderate-intensity PA, (3) walking, and (4) sitting time (as an indicator of sedentary behavior). A continuous IPAQ-score was calculated as total metabolic equivalent of task (MET)-minutes per week = MET level (i.e., walking = 3.3 METs, moderate-intensity PA = 4.0 METs, and vigorous-intensity PA = 8 METs) × minutes of activity × days per week.

### 2.4. Survey Distribution and Data Collection

Parts 1 and 2 were translated by the study group into Danish (N.L.), English (N.L.), Spanish (E.S.), and Dutch (W.K., N.C.V.). Validated translations of the IPAQ-SF were downloaded in the four languages from the official IPAQ webpage [15]. The survey parts were assembled and set up as four web-based surveys (English, Danish, Spanish, and Dutch) using the software REDCap (©2018 Vanderbilt University, Nashville, TN, USA). The survey’s opening question sought to seek consent, which was required to proceed. An invitation letter including a link to the web-based survey was distributed to patients with McArdle disease in Denmark, the Netherlands, and Spain by secure email through the neuromuscular clinics in the respective affiliations. Along with the direct invitation strategy, the patient organization, IamGSD, posted the invitational letter in English and Spanish on their web pages. The letter included a description and aim of the different survey parts; it was highlighted that the survey target group was all adults with a McArdle disease diagnosis and that the survey was also relevant to patients that had never tried or heard of a LCKD. All responses to the survey were automatically stored in a secure REDCap database.

### 2.5. Data Presentation

All data from the four surveys (English, Danish, Spanish, and Dutch) were aggregated into one database and reviewed by one investigator (N.L.). Continuous variables are shown as mean and standard deviation (SD). Non-normal distributed continuous variables are presented as the median and interquartile range (IQR), and categorical variables as frequency and percentages. Normality was assessed visually by histograms. The Pearson chi-square (χ^2^) test was applied to compare the difference in categorical data. A *p*-value ≤ 0.05 was considered significant. All data analyses were carried out using Statistical package for social sciences (SPSS, IBM, version 25) and Microsoft Excel (Microsoft, 2016).

## 3. Results

### 3.1. Study Cohort

We invited 21, 47 and 158 patients with McArdle disease through the Danish, Dutch and Spanish neuromuscular referral clinics, respectively. Furthermore, patients were recruited via IamGSD. A total of 200 responses were collected. Of the 200 responses, 17 patients were excluded due to incomplete or double responses, resulting in 183 responses included in the data analyses. Of the 183 responses, 179 respondents completed all survey parts, and 4 almost completed the survey. We collected 110 responses via IamGSD (English version); and the rest through direct invitation via the Spanish (*n* = 33), Dutch (*n* = 28) and Danish clinics (*n* = 12). The response rates via the Spanish, Dutch and Danish clinics were 20.9%, 59.6% and 57.1%, respectively. All included respondents gave written consent to participate in the study.

The study included individuals from 18 countries and aged between 17–82 years (Table 1). The population was divided into two subgroups: one group that had never tried (subgroup: no LCKD) and one group that had tried a LCKD or variation thereof at least once (subgroup: LCKD). Table 1 shows the patient demographics for the entire population and the two subgroups. Most of the respondents (67.2%) had never tried any kind of a LCKD, whereas 32.8% had tried a LCKD or a variation thereof at least once, but only 13.1% were currently on this type of diet at the time of completing the survey.

**Figure 1:**
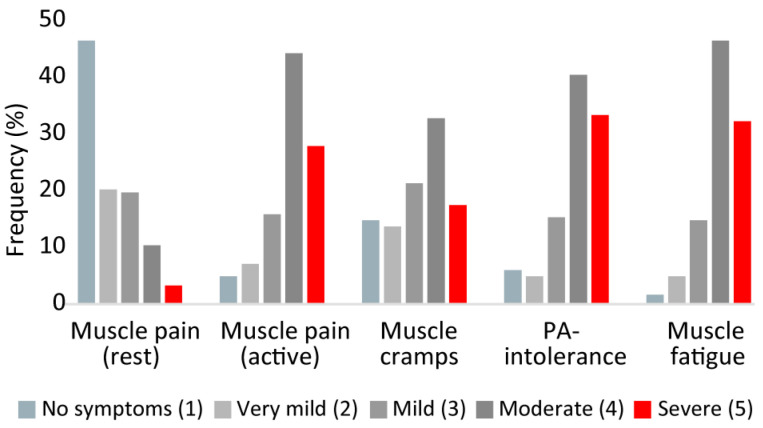
Distribution of McArdle disease-related symptoms. PA: physical activity. Based on *n* = 183 responses.

The patient-reported comorbidities are shown in Table 1. Anxiety and/or depression, followed by chronic pain, were the most common comorbidities. The patient-reported severity of the chosen core symptoms is shown in Figure 1. The derived summarized symptom scores ranged from 7–25. The median BMI in the overall population was 25.9, and the interquartile range was 22.8–30.2 (Table 1). Six (3.3%) patients were underweight (BMI < 18.5), 71 (38.8%) had normal weight (BMI = 18.5–24.9), 57 (31.1%) were overweight (BMI = 25.0–29.9), and 47 (25.7%) patients were obese (BMI > 30) [16]. The two subgroups were very similar with regard to age, sex, BMI, symptom score, and comorbidities. Country of residence varied in the two subgroups, with the most pronounced examples being Spain and the Scandinavian countries (Denmark, Sweden, and the Faroe Islands). Only one of the 33 Spanish patients had tried the diet, vs. 11 of 14 patients from Scandinavia.

One-hundred and seventy-two patients responded to the survey part on PA levels. We did a quality check on the collected data and excluded unrealistic scores. Of the 172 responses, four were excluded from the analyses because of unrealistic high scores (18,944–36,950 MET-minute/week), resulting in 168 responses presented in Table 1. We found numerically higher IPAQ-SF scores in the no-LCKD subgroup compared to the LCKD subgroup.

In the no LCKD subgroup, 63.9% had no plans to start the diet, 33.6% answered ‘maybe’, and 2.5% were planning to start the diet (based on 119 responses). Among the respondents not planning to start the diet, the main reasons were ‘I like my current diet’ (46.1%), ‘complicated to follow’ (14.5%), ‘unappetizing’ (13.2%), and ‘never heard of the diet before’ (15.8%). Less frequent reasons were: ‘complicated in a family with kids’ (6.6%), ‘time-consuming’ (7.9%), and ‘economic reasons’ (3.9%). On the other hand, 28.9% responded with ‘other reasons’, which included; ‘I think it is not healthy’, ‘too much fat’, or ‘I have learned to live with my McArdle disease’.

Among the 60 patients in the LCKD subgroup, half of them had heard about the diet from other patients (50.0%), patient organizations (36.7%), social media including Facebook (25.0%), friends and family (21.7%), or by participation in a research study (18.3%). Only three respondents (5.0%) had heard about the diet from their physicians, and nine patients (15.0%) noted ‘other’.

### 3.2. Experiences with the LCKD

**Figure 2:**
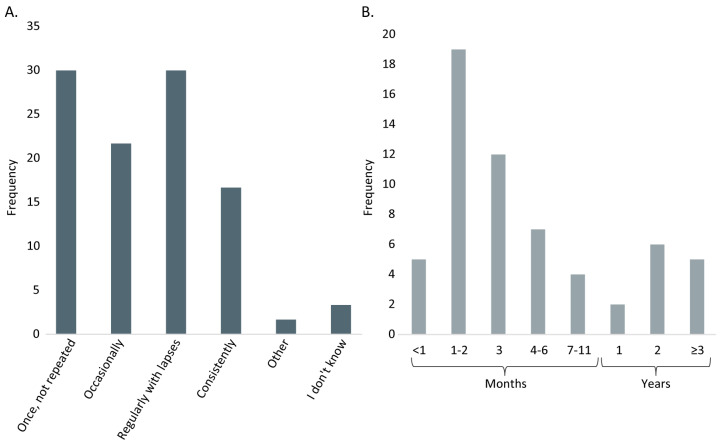
Use of the low carbohydrate ketogenic diet. (**A**) Consistency of use and (**B**) The longest time on the diet. Based on *n* = 60 responses.

The experiences reported by the LCKD subgroup are presented in this and subsequent sections. This subgroup reported that the most important reasons to start the diet were: ‘to improve PA tolerance’ (66.7%), ‘to reduce muscle pain’ (63.3%), ‘to get more energy’ (60.0%), ‘to be less tired’ (50.0%), and ‘to lose weight’ (56.7%). ‘Improvement of mental clarity’ was less frequently cited as a reason to begin a LCKD (16.7%). Two patients noted that they started due to participation in a research trial, and one noted, ‘I don’t know’. The most common LCKD variation that the respondents had adapted to was a modified ketogenic diet containing 60–89% fat (56.6%). Of the respondents, 17 (28.3%) reported they had tried the classical ketogenic diet (containing ≥90% fat), 15 (25.0%) reported trying a low carbohydrate high-fat diet, 3 (5.0%) the ‘one meal a day’ diet and lastly 7 (11.7%) replied ‘other LCKD versions’ that were not listed in the response options. The way the respondents used the LCKD varied as well (Figure 2A). The time patients were on a LCKD without breaks also varied, from under one month to more than three years (Figure 2B). Twenty-eight (47.5%) patients replied ‘yes’ to having used fasting to help transition into ketosis, and of them, 85.7% found it helpful. Twenty-one (35.6%) patients replied ‘yes’ to having used exercise to help transition into ketosis, and of them, 66.7% found it helpful. Fewer patients (18.6%) had used supplements to help transition into ketosis, including MCT-oil (Medium-chain triglycerides oil) (*n* = 6), ketone salts or esters (*n* = 3), high fat, low carb shakes (*n* = 4), special oil (*n* = 1), and pH-corrected Kre-Alkalyn (*n* = 1).

#### 3.2.1. Patient-Reported LCKD Effects

**Figure 3:**
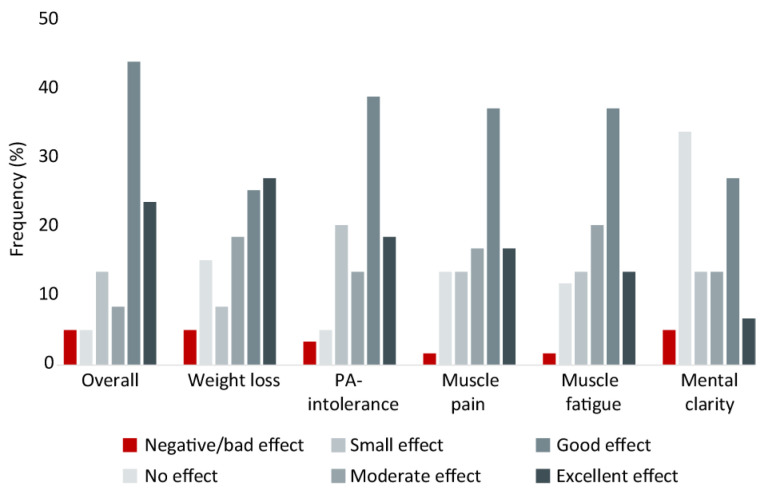
Patient-reported effects of a low-carbohydrate ketogenic diet. PA: physical activity. Based on *n* = 59 responses.

Table 2 presents quotes from selected respondents reflecting different experiences with a LCKD. Most respondents, 67.8%, reported overall good-excellent effects, 22.0% reported overall small-moderate effects, and 10.2% a negative or no effect of a LCKD (Figure 3). The respondents especially reported good-excellent effects on the disease core symptoms: PA tolerance, muscle pain, and muscle fatigue (Figure 3). For all three symptoms, more than 50% reported good-excellent effects, and more than 30% reported small-moderate effects. By contrast, only a minor group of the respondents reported negative effects (1.7–3.4%) or no effect at all (5.1–13.6%). Most respondents also experienced weight loss (Figure 3). Regarding effects on mental clarity, 27.1% reported small-moderate effects, 33.9% had good-excellent effects, and 39% had either no effect or negative effects. 

**Figure 4:**
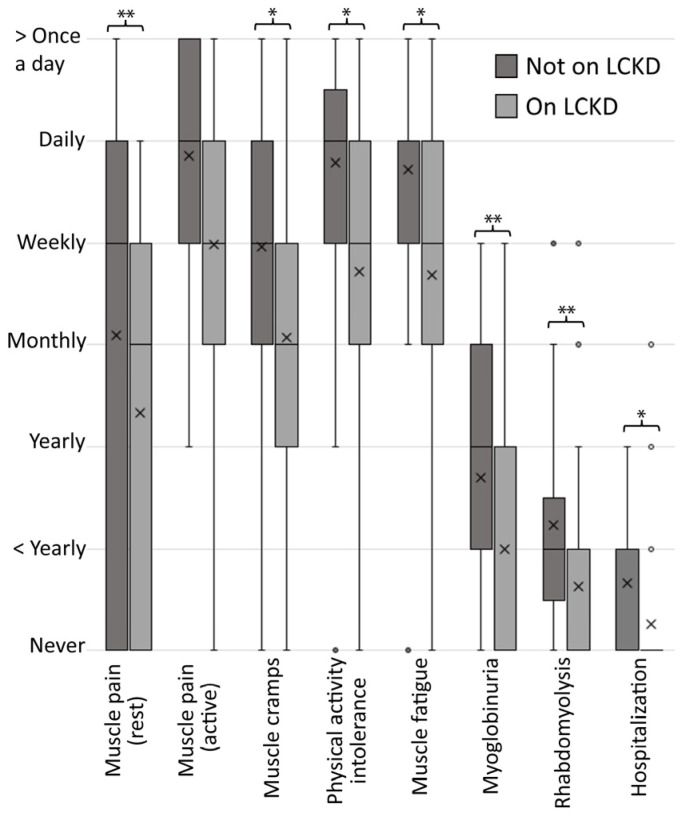
Frequency of McArdle disease-related symptoms while on and not on a low carbohydrate ketogenic diet (LCDK). The boxes illustrate the lower quartiles (Q1), medians (Q2), and upper quartiles (Q3). The cross illustrates the means. The whiskers illustrate the minimum and maximum, and the circles illustrate outliers. Statistically significant differences are illustrated with *: *p* < 0.05 and **: *p* < 0.001. Based on *n* = 57 responses.

Additionally, the respondents graded the frequency of McArdle core symptoms while on and not on a LCKD (Figure 4). The results show that, as far as the respondents recalled, all symptoms occurred less frequently when on a LCKD compared to a period off the diet. The results from the categorical analyses (χ^2^ test) showed that this difference was significant (*p* < 0.05) for all symptoms except “muscle pain (active)” (*p* = 0.068).

To the question, ‘while in ketosis, are your everyday symptoms related to McArdle disease improved?’ 1.8% reported that all symptoms related to McArdle disease had disappeared, 36.8%, 31.6%, 19.3% and 3.5% reported very high, moderate, very small, or no improvement, respectively, and 7.0% indicated ’I don’t know’ (based on 57 respondents). To the question ‘while in ketosis, do you feel as if you are in permanent “second wind”?’ 15.5% of the 58 respondents reported: ‘Yes, all the time’; 43.1%: ‘Yes, but it comes and goes’; 8.6%: ‘Maybe’; 25.9: ‘No’; and 6.9%: ‘I don’t know’.

#### 3.2.2. Consults and Self-Reported Objective Findings

Fifty-five percent of the respondents who tried a LCKD consulted a physician or dietician regarding the LCKD, and of them, 72.7% found the consultation useful, and 27.3% did not. Fifty-two percent had blood cholesterol levels measured (22.6% before starting a LCKD and not repeated thereafter); 19.4% and 22.6% reported that their cholesterol level either lowered or was unchanged, respectively; 22.6% reported that their blood cholesterol level increased while on a LCKD; and the remaining 12.9% replied, ‘I don’t know’. The LCKD is a popular diet for losing weight. To that effect, 81.7% replied they lost weight on a LCKD, one respondent (1.7%) reported weight gain, 15.0% reported no change, and one (1.7%) replied ‘I don’t know’. Blood pressure (BP) was measured in 56.7%, of whom 14.7% only got BP measured before starting the LCKD. The majority reported unchanged BP (55.9%), 8.8% reported elevated and 8.8% lowered BP, and the remaining 11.8% replied, ‘I don’t know’. Out of 59 respondents, 37.3% and 28.8% replied that there was a high chance that they reached ketosis based on results from home tests with either urine sticks or blood tests, respectively. Twelve percent replied that there was a high chance based on symptoms alone. Finally, four respondents replied that they were unlikely to have reached ketosis based on symptoms and one based on home tests. The remaining replied, ‘I don’t know´.

#### 3.2.3. Adverse Effects

**Figure 5:**
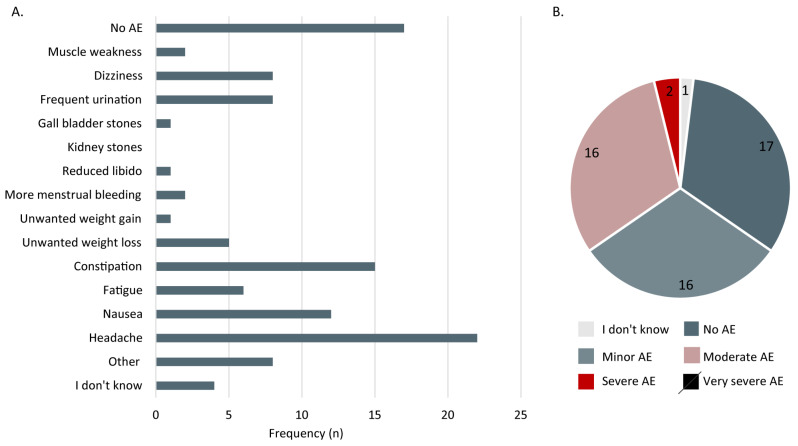
Prevalence of adverse effects (**A**) (*n* = 57) and severity of adverse effects (**B**) (*n* = 52) experienced on a low carbohydrate ketogenic diet. AE: adverse effects; n: number of patients.

The adverse effects are presented in Figure 5. Almost 30% reported no adverse effects from a LCKD. The most common adverse effects were headaches (38.6%), constipation (26.3%), nausea (21.1%), dizziness (14%), and frequent urination (14%) (Figure 5A). The adverse effects were graded as minor or moderate (Figure 5B). Only two patients reported severe adverse effects (one wrote night cramps in the legs, the other marked gall bladder stones), and no one reported very severe adverse effects.

#### 3.2.4. Effects vs. Efforts

**Figure 6:**
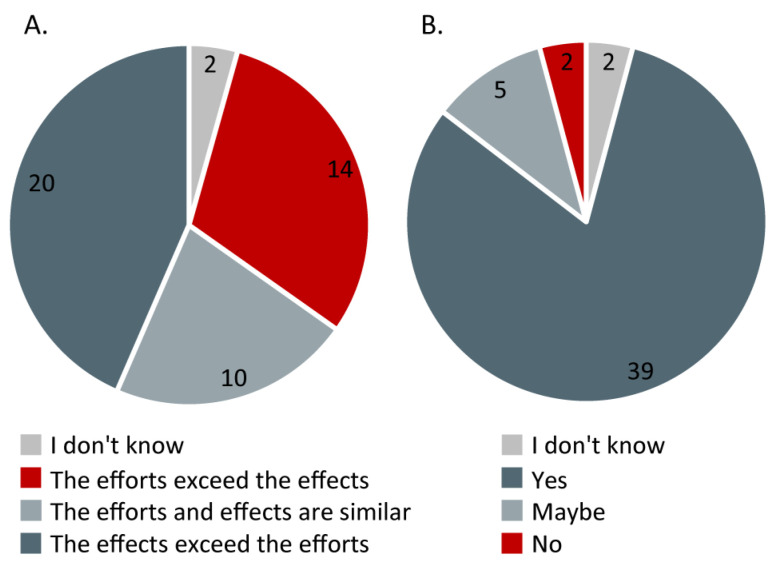
The balance between the efforts versus effects of the low carbohydrate ketogenic diet on overall functioning (**A**) and the tendency to recommend the low carbohydrate ketogenic diet to other individuals with McArdle disease (**B**). Based on *n* = 48 responses.

Because of an inadvertent error, the Danish version of the survey did not include the question on effects vs. efforts and the questions on diet recommendation (see full survey outline in the supplements, questions 31 and 32). Therefore, only 48 of 60 responded to those two questions. Forty-two percent of the respondents reported that the effects of the LCKD exceeded the efforts, 20.8% that the effects and efforts were balanced, and 29.2% that the efforts exceeded the effects (Figure 6A). On the other hand, 81.3% would recommend the diet to other individuals with McArdle disease, 10.4% responded ‘maybe’, and only 4.2% would not recommend the diet to individuals with McArdle disease (Figure 6B).

## 4. Discussion

In this web-based survey, 183 individuals with McArdle disease reported their own experiences related to a LCKD. We found that one-third of this cohort from different countries and continents had tried this dietary approach, with the vast majority (nearly 90%) experiencing some degree of improvement while on a LCKD diet.

Given the lack of strong evidence for a LCKD in this population, the proportion of patients that had tried this type of diet was surprisingly high. One reason for this result could be selection bias, where patients were more prone to reply if they had tried a LCKD, even though the invitation letter highlighted that the survey was relevant to all individuals with a McArdle disease diagnosis. Nonetheless, the frequency is still high, even though there may have been instances of selection bias, and this emphasizes the importance of research in this field.

It is generally described that middle-aged women are more likely than young men to engage in a special diet and that the percentage of adults trying a special diet increases with body weight [17]. Yet, in our study population, neither age, sex, nor BMI seemed to be associated with starting a LCKD, as the two subgroups were very similar. Country of residence, however, showed to differ in the two subgroups. This finding makes good sense, as this study shows that patients primarily heard about the diet from other patients (50%) or their patient organization (37%) and very seldom from their physicians (5%). This would suggest that patients are influenced by what is the trend among other patients in their immediate community and in the patient networks they connect with. To illustrate, only one respondent from Spain in our study had adopted a LCKD, likely indicating that there is no LCKD trend among Spanish individuals with McArdle disease. By contrast, in the Scandinavian population, most respondents had tried the diet (78.6%) initially due to participation in research projects exploring the LCKD in this cohort [3,18], which is why these data need to be interpreted with caution. One could speculate that physically active people could be more prone to adopt a new diet regime. In this study, however, we found a trend toward higher IPAQ scores in the no LCKD subgroup. This result should be interpreted with caution because the IPAQ-SF typically overestimates PA [14]. Indeed, the respondents in our study scored quite highly, with a median of ~2600 MET-min/week of moderate-vigorous PA (i.e., well above the minimum international PA recommendations of 600–1200 MET-min/week) [19]. For instance, only half of the Spanish patients with McArdle disease have been previously reported to meet the minimum international guidelines of PA [20]. Another explanation could be that individuals with McArdle disease perceive their PA as higher because of the PA-induced symptoms, resulting in a higher IPAQ score compared to healthy individuals. Future investigations should include objective measurements of PA (e.g., with accelerometers). The reported comorbidities in the present study were equally distributed in the two subgroups, and besides the high frequency of patients reporting the comorbidity ‘anxiety and/or depression’, the rest of the reported comorbidities were in line with frequencies reported in the EUROMAC registry study [21].

Given the findings of this study, it is not surprising that LCKDs are a topic of considerable interest in the community of individuals with McArdle disease. Our study indicates that the vast majority (89.9%) of the respondents that utilized a LCKD reported some degree of overall beneficial effects, and the majority likewise reported improvements in the core symptoms: PA intolerance, muscle pain and/or fatigue, and that symptoms occurred less frequently compared to when not on a LCKD. Furthermore, most respondents (~80%) reported weight loss, which is warranted in this cohort, where many are overweight [21]. Being overweight can restrict PA, which again can exacerbate symptoms and vice-versa. Less PA can cause weight gain, and in that effect alone, the LCKD could have its place as a weight loss diet in this cohort.

McArdle disease often affects individuals during all tasks involving muscle activation. Thus, symptoms are not only restricted to strenuous exercise, and indeed many individuals report that their symptoms related to activities of daily living (ADL, e.g., house-holding tasks) are most disabling. Therefore, we asked if everyday symptoms related to McArdle disease improved while in ketosis, and approximately 90% reported that their symptoms did improve to varying degrees, in contrast to only 3.5% reporting no improvement. This survey, however, did not include any questions addressing ADL in more detail, which could have been interesting. Quotes number 1 and 4 from selected respondents illustrate some examples of ADL improvement with a LCKD (Table 2). To date, a validated patient-reported outcome measure (PROM) to report on McArdle disease severity and symptoms has not been developed. Such a validated PROM that addresses McArdle disease core symptoms and symptoms during everyday tasks is warranted.

The “second wind” phenomenon is pathognomonic for McArdle disease, and interestingly more than half of the respondents reported that while on a LCKD, they felt as though they were in constant “second wind”, either all the time (15.5%) or periodically (43.1%). The patient organization (IamGSD) published the situation report ‘Nutritional ketosis in McArdle disease’ [4], where 69% of respondents stated they felt as always in “second wind” and 76% reported improved exercise/PA tolerance, which is in line with the present findings.

Conclusively, there is no doubt that most respondents report positive effects of the LCKD, but the question remains—what is causing these effects? In this survey, the patient-reported reasons to start the diet were to improve PA tolerance, reduce muscle pain, and get more energy. One could speculate that the patient-reported diet effects are the same as the ones they expected and that the effects reported could be a placebo effect. This survey can not answer this question and therefore calls for controlled trials. There could also be another case of selection bias, in that the respondents that have experienced positive effects with the diet could be more prone to accept and reply to a survey than those that have experienced negative effects. In the present survey, we received varying response rates from the three referral centers, with as low as 20.9% from the Spanish referral center, which highlights the selection bias issue. Survey studies, in general, have many sources of error, aside from selection bias, including information bias and social desirability bias. Social desirability bias, where respondents skew self-reports in a positive direction, is probably less prominent in this study, as it has been demonstrated to be less pronounced in an online setting [22,23]. The most important information bias that could be present in this study is recall bias, as respondents may have difficulties recalling their symptoms exactly from different periods. Furthermore, this survey cannot control if the respondents were on the LCKD diet and if they reached ketosis. Therefore, future clinical trials exploring the effects of a LCKD should test for diet compliance, including both objective measurements (e.g., plasma ketones) along with diet diaries. The respondents in the present study followed different diet regimes. Future clinical trials should test the same macro-nutritional diet composition for all trial participants.

Nonetheless, the findings in this study, showing remarkable self-reported beneficial effects of the LCKD in a relatively large cohort for a rare disease such as McArdle disease, are of interest and warrant future placebo-controlled trials testing the LCKD potential and effects. To date, only two case reports and one open-label pilot study have been published on LCKD and McArdle disease, all indicating effects of a LCKD on McArdle disease-related symptoms in line with the findings presented in this study [1,2,3]. Based on these studies and the present study, we cannot determine whether the diet effects are placebo, weight loss-associated, and/or actual metabolic effects related to enhanced ketone body or fat oxidation. Future clinical trials must be performed to clarify what mechanisms contribute to the patient-reported effects shown here. The findings from this study suggest incorporating PA tolerance, especially pre-second wind, as an outcome measure. Furthermore, it is warranted to include PROMs: ideally, a McArdle disease-specific PROM that includes the McArdle disease core symptoms and questions on ADL.

Interestingly, only 40% (*n* = 24) of the LCKD subgroup were currently on a LCKD. This was surprising when so many found the diet effective. One reason could be the effort it takes to stick to a LCKD. This type of diet is indeed invasive to social life in a family. It is time-consuming, changes living habits and cooking routines, and indeed it has been reported in other disease cohorts that it is a hard diet to follow—especially in the long run [24]. In our study, 50% reported that effects and efforts were either balanced (29%) or that efforts exceeded the effects (29%). Some of these efforts are emphasized in some of the selected experiences presented in Table 2. Another reason could be adverse effects. However, in this study, adverse effects were mostly graded as minor and, therefore, perhaps not as relevant for maintaining the diet. Common symptoms that occur when starting a LCKD are nausea, headache, fatigue and/or diarrhea/constipation. These symptoms are also known as the ‘keto-flu’. The ‘keto flu’ symptoms are transient and can be avoided or minimized with a slow up-titration of the diet. In the present study, we assessed if an adverse effect had occurred, but we did not assess the time frame of the occurrence. Thus, we can only assume that some of the presented adverse events were simply transient ‘keto-flu’ symptoms. The most investigated LCKD regime is the classical ketogenic diet in cohorts of children with epilepsy. Long-term effects of more liberate LCKD regimes in adult populations are not well studied and are non-existent in McArdle disease. Long-term effects and adverse effects need to be investigated in future studies. The classical ketogenic diet probably causes more side effects. We propose investigating more liberate LCKD regimes, planned by trained dieticians to ensure the healthiest possible diet, in future trials. Lastly, a reason could be the lack of scientific evidence of the LCKD effects and the lack of support from the healthcare systems to help maintain and design a proper LCKD regime. Indeed, dietetic guidance has been shown to heighten compliance [24].

## 5. Conclusions

A third of the 183 respondents with McArdle disease that we studied had utilized a LCKD, and almost 90% of them experienced some degree of symptom improvement with the diet, with prominent effects on disease-related core symptoms (e.g., PA intolerance, muscle pain, and muscle fatigue). Adverse effects were rare overall and likely related to the common experience of transitioning to a LCKD. These findings underline the patient-led demand and need for well-designed clinical trials to examine the therapeutic potential of a LCKD in McArdle disease, both short- and long-term, and more specifically, to better understand if a LCKD elicits a metabolic workaround for impaired glycolytic muscle metabolism.

## Figures and Tables

**Table 1:** Demographics.

	Overall Sample *n* = 183	Subgroup: No LKCD *n* = 123 (67.2%)	Subgroup: LCKD *n* = 60 (32.8%)
Age, years (median (IQR))	*n* = 178 51 (37–63)	*n* = 123 51 (37–65)	*n* = 57 52 (38–60)
Sex (*n* (%))	*n* = 183	*n* = 123	*n* = 60
Female	108 (59.0%)	73 (59.3%)	35 (58.3%)
Male	74 (40.4%)	50 (40.7%)	24 (40.0%)
Other	1 (0.5%)		1 (1.7%)
BMI, kg/m^2^ (median (IQR))	*n* = 181 25.9 (22.8–30.2)	*n* = 121 25.5 (22.2–30.1)	*n* = 60 27.0 (23.2–30.6)
McArdle symptom score (median (range; IQR))	*n* = 183 18.0 (7–25; 15–20)	*n* = 123 18.0 (7–25; 15–20)	*n* = 60 18.0 (9–25; 15–20)
Country (*n* (%_subgroup_)(%_country_))	*n* = 183	*n* = 123	*n* = 60
DK + FO + S	14 (7.7%)	3 (2.4%) (21.4%)	11 (18.3%) (78.6%)
ES	33 (18.0%)	32 (26.0%) (97.0%)	1 (1.7%) (3.0%)
NL	28 (15.3%)	23 (18.7%) (82.1%)	5 (8.3%) (17.9%)
USA + CAN	47 (25.7%)	24 (19.5%) (51.1%)	23 (38.3%) (48.9%)
UK + IRL	30 (16.3%)	24 (19.5%) (80.0%)	6 (10.0%) (20.0%)
AUS + NZ	14 (7.7%)	8 (6.5%) (57.1%)	6 (10.0%) (42.9%)
Other countries	17 (9.3%)	9 (7.3%) (52.9%)	8 (13.3%) (47.1%)
IPAQ-SF, MET-min/week (median (ICR))	*n* = 168 2560 (1188–4617)	*n* = 111 2880 (1356–5295)	*n* = 57 2079 (922–3795)
Comorbidities (*n* (%))	*n* = 183	*n* = 123	*n* = 60
Anxiety/depression	74 (40.4%)	49 (39.8%)	25 (41.7%)
Chronic pain	44 (24.0%)	29 (23.6%)	15 (25.0%)
Rheumatoid arthritis	7 (3.8%)	5 (4.1%)	2 (3.3%)
Osteoarthritis	12 (6.6%)	4 (3.3%)	8 (13.3%)
Chronic lung disease	15 (8.2%)	8 (6.5%)	7 (11.7%)
Severe heart disease	14 (7.7%)	7 (5.7%)	7 (11.7%)
Hypertension	30 (16.4%)	12 (9.8%)	18 (30%)
Hypercholesterolemia	29 (15.8%)	14 (11.4%)	15 (25.0%)
Diabetes	20 (10.9%)	18 (14.6%)	2 (3.3%)
Liver/gut-disease	7 (3.8%)	4 (3.3%)	3 (5.0%)
Cancer or cancer related	6 (3.3%)	5 (4.1%)	1 (1.7%)
Other	26 (14.2%)	9 (7.3%	11 (18.3%)
None	29 (15.8%)	17 (13.8%)	12 (20%)
‘I don’t know’	18 (9.8%)	17 (13.8%)	1 (1.7%)

Data are frequency (percentage). Abbreviations: BMI, body mass index; LCKD, low carbohydrate ketogenic diet; IQR, interquartile range; IPAQ-SF, international physical activity questionnaire short form; MET, metabolic equivalent of task. Countries: AUS, Australia; CAN, Canada; DK, Denmark; FO, Faeroe islands; IRL, Ireland; NL, The Netherlands; NZ, New Zealand; SP, Spain; ES, Sweden; UK, United Kingdom; USA, United States of America. Other countries: Belgium, France, Germany, Italy, Kuwait, Malta, Philippines.

**Table 2:** Quotes from selected respondents.

#1	“Importantly, there are still times when I need to get into second wind, even on the ketogenic diet during exercise. But getting into second wind feels faster and smoother while following a ketogenic diet and I usually don’t have to stop and rest prior to getting into second wind (but I may slow down, for example, prior to getting into second wind). But in terms of activities of daily living, the ketogenic diet makes all my symptoms of McArdle’s go away—things like walking across a parking lot, drying my hair with a blow dryer, lifting things, etc. All of those things become easy and normal. But when actually doing exercise, such as quick walking, biking or walking uphill, I still need to get into second wind. But being on the ketogenic diet makes this easier and less painful.”
#2	“I have been and continue to be a strong advocate of keto/low carb for McArdle disease—it allows us to do things we thought not possible. I never dreamed I would be able to jog for 50 min non-stop, yet in the past when in ketosis I have done so…”
#3	“Some of my improvement in symptoms was due to weight loss, 14.5 Kg. Being diagnosed at age 76 my normal diet was deeply ingrained and although I felt better in ketosis, I preferred to keep eating what I always had.”
#4	“A LCKD is very helpful in reducing pre-second wind symptoms thereby making ADLs much easier. I am also able to do more aerobically—push the anaerobic threshold further. It is not always easy to maintain a LCKD, but the effects are well worth it.”
#5	“I was on a 3-month period of Keto diet. I went on a family vacation and was amazed at my ability to walk as much as 15 K steps without pain or cramping.”
#6	“Hard to maintain compliance without putting a lot of effort into shopping and meal preparation.”
#7	“It would be so much easier to stay on a ketogenic diet if the resources were cheaper and more readily available. You really have to enjoy cooking from scratch on a keto diet—and sometimes it would just be nice to have crunchy food.”
#8	“Not sure if the small improvement effects I felt when I was on ketonic diet were real or due to placebo effect.”
#9	“Keto diet is expensive to maintain but worth the effort to me. Reduced daily pain, cramping, and quicker recovery from episodes of cramping and fatigue.”
#10	“I found for me, I couldn’t do it permanently as it was too restricted and I was getting sick of the same foods.”
#11	“The efforts exceeded the effects by a small margin. I definitely saw a solid reduction in McArdle symptoms. The most apparent being that I did not feel as if I needed to stop and rest before the second wind came on. I would say that it smoothed out the curve. I would still get a little tired before the second wind came on but the transition was more subtle, and I could last longer doing an activity.”
#12	“Diet helped very much, I benefited very much. Only maintaining it without the support of a dietician is very difficult. It requires a lot of discipline. Also, a lot of foods contain carbohydrates. The same goes for vegetables. It’s really hard to find what you can and cannot eat.” (Translated from Dutch)
#13	“I notice a very big difference in daily things and with training. However, activities that take a lot of strength are still too heavy. So it helps me a lot with aerobic efforts but anaerobic efforts remain heavier” (Translated from Dutch)

## Data Availability

Most data supporting this study are presented in this manuscript. Due to ethical concerns and GDPR regulations, supporting data cannot be made openly available. Data can be shared under some conditions upon request.

## Supplementary Materials

The following supporting information can be downloaded at: https://www.mdpi.com/article/10.3390/nu15040843/s1, File S1: 1. General questions about you and your disease, File S2: 2. Questions about your diet and experience with a ketogenic diet.
