# Supplementary material for: Patient-Reported Experiences with a Low-Carbohydrate Ketogenic Diet: An International Survey in Patients with McArdle Disease
Source: Nutrients. 2023 Feb 7;15(4):843. doi: 10.3390/nu15040843 (PMC9964801; doi:10.3390/nu15040843)
Supplement: Supplementary file 1 [file nutrients-15-00843-s001.zip › File S1.pdf]

1. General questions about you and your disease

Thank you for participating in this survey "Ketogen diet for patients with McArdle disease - a survey study". The survey consists of 6 sections. When you have answered all the questions in one section, click on "submit" and you will automatically move on to the next section. When you have clicked "submit" you cannot return to the previous questions. It takes approx. 30 minutes to answer the entire survey.

In the first section of the survey, there are some general questions about you including questions regarding McArdle disease. Next, there will be a section on your diet, including a specific section on the ketogenic diet. It is important that you answer the entire survey, even if you have not followed a ketogenic diet. Finally, there will be some general sections on physical activity, fatigue, sleep, level of functioning, and quality of life.

---

1. Informed consent: I am 18 years old or older, and I wish to participate voluntarily in the following survey. I am aware that I can withdraw my consent at any time. The results from this survey will be published anonymously, and I am informed that any personal information about me will be left out.

☐ I consent

---

2. Your initials

____

---

3. Gender?

◯ Female ◯ Male ◯ Other

---

4. Date of birth?

____

---

5. Todays date?

____

Age

____

---

6. Have you been diagnosed with McArdle disease?

◯ Yes
◯ Maybe
◯ No
◯ I am currently beeing examined for McArdle disease. Diagnosis not yet confirmed.

7. How many years have you been diagnosed with McArdle disease?
(Click on the arrow - and select the best answer)

◯ Less then 1 year ◯ 1 year ◯ 2 years ◯ 3 years ◯ 4 years ◯ 5 years ◯ 6 years
◯ 7 years ◯ 8 years ◯ 9 years ◯ 10 years ◯ 11 years ◯ 12 years ◯ 13 years
◯ 14 years ◯ 15 years ◯ 16 years ◯ 17 years ◯ 18 years ◯ 19 years ◯ 20 years
◯ 21 years ◯ 22 years ◯ 23 years ◯ 24 years ◯ 25 years ◯ 26 years ◯ 27 years
◯ 28 years ◯ 29 years ◯ 30 years ◯ 31 years ◯ 32 years ◯ 33 years ◯ 34 years
◯ 35 years ◯ 36 years ◯ 37 years ◯ 38 years ◯ 39 years ◯ 40 years ◯ 41 years
◯ 42 years ◯ 43 years ◯ 44 years ◯ 45 years ◯ 46 years ◯ 47 years ◯ 48 years
◯ 49 years ◯ 50+ years

---

8. How were you diagnosed with McArdle disease?

☐ Diagnosis was made after a random discovery of elevated blood creatine kinase (CK)
☐ Screening after positiv diagnosis of a sibling or family member
☐ Diagnosis was confirmed by a cycle- or handgrip test
☐ Diagnosis was confirmed by muscle biopsy
☐ Diagnosis was confirmed by DNA test
☐ Other
☐ I don't know
(Click one or more boxes)

If you selected the option "Other", please elaborate:

____

---

9. Do you have any relatives, who are diagnosed with McArdle disease?

☐ No
☐ One or more siblings
☐ My mom
☐ My dad
☐ My grandparents
☐ One or more of my cousins
☐ My child/children
☐ Other
☐ I don't know
(Click one or more boxes)

10. How old were you, when you first started noticing symptoms of McArdle disease?
(click on the arrow - and select the best answer)

◯ I have had symptoms for as long as i can remember ◯ 1 year old ◯ 2 years old ◯ 3 years old
◯ 4 years old ◯ 5 years old ◯ 6 years old ◯ 7 years old ◯ 8 years old ◯ 9 years old
◯ 10 years old ◯ 11 years old ◯ 12 years old ◯ 13 years old ◯ 14 years old ◯ 15 years old
◯ 16 years old ◯ 17 years old ◯ 18 years old ◯ 19 years old ◯ 20 years old ◯ 21 years old
◯ 22 years old ◯ 23 years old ◯ 24 years old ◯ 25 years old ◯ 26 years old ◯ 27 years old
◯ 28 years old ◯ 29 years old ◯ 30 years old ◯ 31 years old ◯ 32 years old ◯ 33 years old
◯ 34 years old ◯ 35 years old ◯ 36 years old ◯ 37 years old ◯ 38 years old ◯ 39 years old
◯ 40 years old ◯ 41 years old ◯ 42 years old ◯ 43 years old ◯ 44 years old ◯ 45 years old
◯ 46 years old ◯ 47 years old ◯ 48 years old ◯ 49 years old ◯ 50+ years old

11. What was your debut symptom(s) of McArdle disease?

☐ I had no symptoms
☐ Muscle pain
☐ Muscle cramps
☐ Muscle fatique
☐ Activity intolerance
☐ Episode(s) of dark colored urin (myoglobinuria)
☐ Episode(s) of rhabdomyolysis
☐ Other symptoms
(Click one or more boxes)

Please elaborate:

____

12. What are your primary symptoms now?

☐ I have no symptoms
☐ Muscle pain
☐ Muscle cramps
☐ Muscle fatique
☐ Activity/Exercise intolerance
☐ Episodes of dark colored urin (myoglobinuria)
☐ Episodes of rhabdomyolysis
☐ Other symptoms
(Click one or more boxes)

Please elaborate:

____

13. Please grade your average McArdle disease symptoms on a scale from no symptoms to severe symptoms. When answering this question, your starting point has to be in a periode, where you were not on a keto-diet.

	No symptoms	Very mild	Mild	Moderate	Severe
Muscle pain (resting)	◯	◯	◯	◯	◯
Muscle pain (active)	◯	◯	◯	◯	◯
Muscle cramps	◯	◯	◯	◯	◯
Activity intolerance	◯	◯	◯	◯	◯
Muscle fatique	◯	◯	◯	◯	◯

14. Please grade how often you experience McArdle symptoms on a scale from never to more than once a day. When answering this question, your starting point has to be in a periode, where you were not on a keto-diet.

	Never	Less than once a year	Yearly	Monthly	Weekly	Daily	More than once a day
Muscle pain (resting)	◯	◯	◯	◯	◯	◯	◯
Muscle pain (active)	◯	◯	◯	◯	◯	◯	◯
Muscle cramps	◯	◯	◯	◯	◯	◯	◯
Activity intolerance	◯	◯	◯	◯	◯	◯	◯
Muscle fatique	◯	◯	◯	◯	◯	◯	◯
Myoglobinuria (dark colored urin)	◯	◯	◯	◯	◯	◯	◯
Rhabdomyolysis	◯	◯	◯	◯	◯	◯	◯
Hospitalization due to McArdle disease	◯	◯	◯	◯	◯	◯	◯

15. Do you know, which specific mutations you have in the McArdle gene (PYGM gene, e.g. R50X)

◯ Yes ◯ No

If yes, please indicate the two causative mutations (example R50X, M1V):

____

If not, are we allowed to contact your medical specialist to collect the information?

◯ Yes ◯ No

If yes, who is your medical specialist: clarify GP or McArdle specialist (where appropriate) (write: your doctors name and telephone, email and/or hospital address)

____

16. Height (cm):

____
(In centimeters, e.g. 173)

17. Weight (kg):

____
(In kilograms, e.g. 67)

BMI

____

18. Home city and country:

____
(eg. London, UK)

19. Your current living situation?	◯ I live alone<br>◯ I live alone with my child/children<br>◯ I live with my partner without children<br>◯ I live with my partner and child/children<br>◯ I live with my spouse without children<br>◯ I live with my spouse and child/children<br>◯ I do not want to answer this question
20. Do you have children?	◯ I do not have kids<br>◯ 1 child<br>◯ 2 children<br>◯ 3 children<br>◯ 4 children<br>◯ More than 4 children<br>◯ I do not want to answer this question
21. What is your highest level of education?	◯ I have not attended school<br>◯ Primary - and Secondary Education<br>◯ Further Education/College<br>◯ Higher Education/University Undergraduate<br>◯ University Postgraduate<br>◯ Other
22. Your current job:	◯ I work full time<br>◯ I work part time<br>◯ Under education/student<br>◯ Temporary sick leave<br>◯ Retired<br>◯ Early retirement<br>◯ Unemployed<br>◯ Other
What type of job do you have?	◯ Stationary job, for example in a office<br>◯ Light physical work, for example in a kindergarten<br>◯ Hard physical work, for eksampel a carpenter
Outside job	________________________________
23. Besides McArdle disease, do you currently have one or more of the following diseases:	☐ Anxiety and/or depression<br>☐ Chronic pain, which effects your physical state/mobility<br>☐ Rheumatoid arthritis<br>☐ Osteoarthritis<br>☐ Chronic Lung Disease (eg. asthma /COPD)<br>☐ Severe heart disease, eg. heart failure or previous heart attack<br>☐ Hypertension<br>☐ Elevated blood-cholesterol<br>☐ Diabetes<br>☐ Liver or gut-disease<br>☐ Cancer related diseases<br>☐ Other diseases<br>☐ I don't know<br>☐ No, I do not have any of the listed diseases<br>(Click one or more boxes)

If you selected the option "Other diseases", please elaborate:

____

24. Current medication:

- ☐ Currently I do not take any medication
- ☐ Medication for anxiety, depression or insomnia
- ☐ Medication for other psychiatric disease
- ☐ Medication for hypertension
- ☐ Medication for elevated cholesterol
- ☐ Medication for diabetes
- ☐ Medication for lung disease (COPD, asthma)
- ☐ Medication for cancer (eg. chemotherapy)
- ☐ Permanent treatment with pain killers
- ☐ Medical cannabis
- ☐ Supplements including vitamins
- ☐ Other medical treatment
- ☐ I don't know

(Click one or more boxes)

If you selected the option "Other medical treatment", please elaborate:

____
