# Supplementary material for: Patient-Reported Experiences with a Low-Carbohydrate Ketogenic Diet: An International Survey in Patients with McArdle Disease
Source: Nutrients. 2023 Feb 7;15(4):843. doi: 10.3390/nu15040843 (PMC9964801; doi:10.3390/nu15040843)
Supplement: Supplementary file 1 [file nutrients-15-00843-s001.zip › File S2.pdf]

2. Questions about your diet and experience with a ketogenic diet

This section contains questions about your diet and your experience with a ketogenic diet or a variation thereof. It is important that you answer all the questions, even if you do not know or have tried a ketogenic diet.

Currently, there is no satisfactory treatment for McArdle disease. The limitation in people with McArdle disease is due to limited availability of energy in the muscle due to affected carbohydrate metabolism. A key element in improving muscle function is therefore to introduce alternative energy sources. Ketone bodies are a well-known source of energy for both brain and muscle. Ketone bodies are produced during fasting or with a ketogenic diet. A ketogenic diet is a diet high in fat and low in carbohydrates. The classic ketogenic diet contains up to 90% fat, but there are several alternative diets with less fat, including a modified ketogenic diet, Atkins diet, LCHF (low carb high fat), OMAD (one meal a day) and more. In this survey we are interested in information on all variations. The rationale for a ketogenic diet for persons with McArdle disease makes good sense in theory, as the diet would contribute with a new source of energy (ketone bodies) independent of the affected carbohydrate breakdown. However, we still lack proof on whether the diet actually works. The purpose of this survey is to report experiences with variants of the ketogenic diet in a group of individuals diagnosed with McArdle disease.

When you have answered all the questions in this section, click on "submit" and you will automatically move on to the next section. It is not possible to save the answer along the way and return later.

---

1. How would you describe the composition of your current diet?

◯ I don't know
◯ Healthy and varied
◯ Unvaried
◯ Fat-based diet (e.g. ketogenic, LCHF, atkins..)
◯ Carbohydrate-based diet
◯ Other specific diet

Please elaborate: ______________________________

---

2. Have you heard about the ketogenic diet?

◯ Yes ◯ No

If yes, where have you heard of the ketogenic diet?

☐ Facebook
☐ My doctor
☐ From a patient organisation (for example IAMGSD)
☐ From other patients with McArdle disease
☐ Family or friends
☐ From participating in a research project
☐ Other
(Click one or more boxes)

If you have selected the option "Other", please elaborate: ______________________________

---

3. Are you currently on the ketogenic diet (or a variation of a ketogenic diet)?

◯ Yes ◯ No

---

4. Have you tried the ketogenic diet before (or a variation of the ketogenic diet)?

◯ Yes ◯ No

5. Are you planning to start the ketogenic diet or a variation of the diet?

◯ Yes ◯ No ◯ I am currently on the ketogenic diet ◯ Maybe

If no, why not? Please select one or more of the reasons listed, and then the survey is complete.

☐ I have not heard of the ketogenic diet before, and I would like to know more about it, before I could consider starting
☐ Economical reasons
☐ The diet is time consuming
☐ The diet is unappetizing
☐ The diet is complicated to follow
☐ The diet is complicated to follow in a family with kids
☐ I like my current diet
☐ Other
(Click one or more boxes)

Please elaborate, why you have not tried the diet, or a variation of the ketogenic diet, before. Or/and why you wish not to start in the future:

6. STOP QUESTION: If you have never been on a ketogenic diet or a variation of the diet, this part of the survey ends and you will automatically be transfered to the next part.

◯ I am not on a ketogenic diet (or a variation of the diet) - stop ◯ I have never been on a ketogenic diet (or a variation of the diet) - stop ◯ I am currently or have been on a ketogenic diet (or a variation of the diet) - The survey continues

7. For how long have you tried the ketogenic diet without breaks?
(Click on the arrow - and select the best answer)

◯ Less than one week ◯ 1 week ◯ 2 weeks ◯ 3 weeks ◯ 4 weeks ◯ 5 weeks
◯ 6 weeks ◯ 7 weeks ◯ 8 weeks ◯ 3 months ◯ 4 months ◯ 5 months ◯ 6 months
◯ 7 months ◯ 8 months ◯ 9 months ◯ 10 months ◯ 11 months ◯ 1 year ◯ 1-2 years
◯ 2-3 years ◯ Over 3 years ◯ I have never tried the ketogenic diet before

8. How often have you been using the ketogenic diet?

☐ Once and not repeated
☐ Occasionally
☐ Regularly, with occasional lapses
☐ Consistently
☐ I do not know
☐ Other
(Click one or more boxes)

Please elaborate:

9. What is the reason that you started / are planning to start the ketogenic diet (or a variation of the ketogenic diet)?

- ☐ To get more energy
- ☐ To improve my physical shape and activity tolerance
- ☐ To lower the risk of muscle pain
- ☐ To lose weight
- ☐ To gain more mental clarity
- ☐ To be less tired
- ☐ Other
- ☐ I do not know

(Click one or more boxes)

If you have selected the option "Other", please elaborate:

---

10. What kind of ketogenic diet are you on / have you followed?

- ☐ I have never tried the ketogenic diet
- ☐ The classical ketogenic diet (90% fat)
- ☐ A modified ketogenic diet / modified Atkins diet (moderate fat 60-90%, low carb)
- ☐ OMAD (one meal a day)
- ☐ LCHF (low carb, high fat)
- ☐ Vegetarian LCKD (low carb, ketogenic diet)
- ☐ Vegan LCKD (low carb, ketogenic diet)
- ☐ The low-carb "1-2-3 Healthy Eating Approach" (1 unit carbs, 2 protein, 3 vegetables)
- ☐ Other

(Click one or more boxes)

Please elaborate:

---

11. Do you know the composition of the three major macronutrients (carbohydrates, protein, fat) in your keto-diet. If yes, please identify the composition that allows you to achieve ketosis:
(Click on the arrow - and select the best answer)

◯ I do not know ◯ 65% fat, 15% protein, 20 % carbohydrate ◯ 70% fat, 20% protein, 10 % carbohydrate
◯ 75% fat, 15% protein, 10 % carbohydrate ◯ 80% fat, 10% protein, 10 % carbohydrate
◯ 80% fat, 15% protein, 5 % carbohydrate ◯ 85% fat, 10% protein, 5 % carbohydrate
◯ 90% fat, 5% protein, 5 % carbohydrate ◯ Other composition

---

12. Have you consulted with a doctor or a dietician, regarding the ketogenic diet, before starting and/or during the diet?

◯ Yes ◯ No

If yes, was it helpful?

◯ Yes ◯ No

---

13. Have you had your blood cholesterol levels measured, before starting and/or during the diet?

◯ Yes ◯ No

If yes, please choose the option that fits best:
(Click on the arrow - and select the best answer)

◯ I do not know ◯ My cholesterol levels have only been measured before I started the diet
◯ My cholesterol levels are lower when I am on the diet ◯ My cholesterol levels are the same (unchanged) when I am on the diet ◯ My cholesterol levels are higher when I am on the diet

14. Have you had your blood pressure measured, before starting and/or during the diet?

◯ Yes ◯ No

If yes, please select the option that fits best:
(Click on the arrow - and select the best answer)

◯ I do not know ◯ My blood pressure has only been measured before I started the diet
◯ My blood pressure is lower when I am on the diet ◯ My blood pressure is the same (unchanged) when I am on the diet ◯ My blood pressure is higher when I am on the diet

15. Has your weight changed while being on a keto-diet?

◯ Yes ◯ No

If yes, select the option that fits best:
(Click on the arrow - and select the best answer)

◯ I do not know ◯ I have only weight myself before I started the diet ◯ I have lost weight during the diet
◯ My weight has stayed the same (unchanged) during the diet ◯ I have gained weight during the diet

16. Have you used a keto cook book for inspiration?

◯ Yes ◯ No

If yes, which:

____

17. Have you been able to get into ketosis (indicated by a change in color on urine dipstick, or by a blood test)?
(Ketosis is the condition the body enters when following a ketogenic diet or when fasting. Ketosis is the result of the body producing ketone bodies. Ketone bodies are a good source of energy for the brain and muscle. Ketone bodies can be measured in blood and in urine. Click on the arrow - and select the best answer)

◯ I do not know ◯ Yes, there is a high chance, based on symptoms, but I have not been tested
◯ Yes, there is a high chance, based on symptoms, but I have not been able to see it on a test
◯ Yes, there is a high chance, based on a change in color on the urine strip ◯ Yes, there is a high chance, based on a blood test (value over 0.5 mmol/L) ◯ No, very unlikely, based on symptoms, but I have never been tested ◯ No, based on urine strip and blood test

18. How long does it take you to get into ketosis?
(Click on the arrow - and select the best answer)

◯ I do not know ◯ within 24 hours ◯ 2 days ◯ 3 days ◯ 4 days ◯ 5 days ◯ 6 days
◯ 7 days ◯ 8 days ◯ 9 days ◯ 10 days ◯ 11 days ◯ 12 days ◯ 13 days ◯ 14 days
◯ 15 days ◯ 16 days ◯ 17 days ◯ Over 18 days ◯ I have not been in ketosis

---

19. Do you use / have you used urine dipsticks to check whether you are in ketosis?

◯ Yes ◯ No

What is the maximum level of ketosis you have reached on the urine strips? (on a scale from 1-5)
(Click on the arrow - and select the best answer)

◯ I do not know ◯ No change in color (no ketones) ◯ A minimal change in color (1)
◯ A small change in color (2) ◯ A medium change in color (3) ◯ A large change in color (4)
◯ A maximal change in color (maximal ketones) (5)

If yes, what is your average level of ketosis, based on a urine strip (on a scale from 1-5)
(Click on the arrow - and select the best answer)

◯ I do not know ◯ No change in color (no ketones) ◯ A minimal change in color (1)
◯ A small change in color (2) ◯ A medium change in color (3) ◯ A big change in color (4)
◯ A maximal change in color (maximal ketones) (5)

---

20. Do you use / have you used blood test (at home) to check whether you are in ketosis?

◯ Yes ◯ No

If yes, what level of ketosis did you maximum reach on the blood test? (Please specify the concentration of ketones in your blood indicated by the device)
(Click on the arrow - and select the best answer)

◯ I do not know ◯ 0,0 to 0,2 mmol/L ◯ 0,3 to 0,4 mmol/L ◯ 0,5 to 0,6 mmol/L ◯ 0,7 to 0,9 mmol/L
◯ 1,0 to 1,2 mmol/L ◯ 1,3 to 1,5 mmol/L ◯ 1,6 to 1,9 mmol/L ◯ 2,0 to 2,5 mmol/L
◯ 2,5 to 3,0 mmol/L ◯ Over 3,0 mmol/L

If yes, what level of ketosis did you on average reach on the blood test (Please specify the concentration of ketones in your blood indicated on the device):
(Click on the arrow - and select the best answer)

◯ I do not know ◯ 0,0 to 0,2 mmol/L ◯ 0,3 to 0,4 mmol/L ◯ 0,5 to 0,6 mmol/L ◯ 0,7 to 0,9 mmol/L
◯ 1,0 to 1,2 mmol/L ◯ 1,3 to 1,5 mmol/L ◯ 1,6 to 1,9 mmol/L ◯ 2,0 to 2,5 mmol/L
◯ 2,5 to 3,0 mmol/L ◯ Over 3,0 mmol/L

---

21. Have you used any supplements to help transition into ketosis?

◯ Yes ◯ No

If yes, please choose which:

☐ MCT-oil
☐ Ketone-salts
☐ Ketone-esters
☐ Triheptanoin oil
☐ Other special oil
☐ Other
(Click one or more boxes)

Please write which supplements you have been taking:

____

22. Have you used fasting to help transition into ketosis?

◯ Yes ◯ No

If yes, for how many hours did you fast?
(Click on the arrow - and select the best answer)

◯ I do not know ◯ 0-6 hours ◯ 7-12 hours ◯ 13-18 hours ◯ 19-24 hours ◯ 25-30 hours
◯ 26-36 hours ◯ 37-42 hours ◯ 43-48 hours ◯ over 48 hours

If yes, did fasting improve your ability to get into ketosis?
(Click on the arrow - and select the best answer)

◯ I do not know ◯ Yes ◯ No

23. Have you used exercise to help transition into ketosis?

◯ Yes ◯ No

If yes, did the exercise improve your ability to get into ketosis?
(Click on the arrow - and select the best answer)

◯ I do not know ◯ Yes ◯ No

24. Please grade the effect of the ketogenic diet (or a variation of the ketogenic diet) on different parameters (on a scale from a negative effect to an excellent effect). When answering this question, your starting point must be when you were on the diet.

	A negative/bad effect	No effect	A small effect	A moderate effect	A good effect	An excellent effect
How is or was the overall effect?	◯	◯	◯	◯	◯	◯
Effects on weight loss	◯	◯	◯	◯	◯	◯
Improved activity tolerance	◯	◯	◯	◯	◯	◯
Less muscle pain	◯	◯	◯	◯	◯	◯
Less muscle fatigue (non active)	◯	◯	◯	◯	◯	◯
Less muscle fatique (active)	◯	◯	◯	◯	◯	◯
Mental clarity	◯	◯	◯	◯	◯	◯

25. Please grade how often you experience McArdle symptoms on a scale from never to more than once a day. When answering this question, your starting point has to be in a periode, where you were on a keto diet.

	Never	Less than yearly	Yearly	Monthly	Weekly	Daily	More than once a day
Muscle pain (resting)	◯	◯	◯	◯	◯	◯	◯
Muscle pain (active)	◯	◯	◯	◯	◯	◯	◯
Muscle cramps (contractures)	◯	◯	◯	◯	◯	◯	◯

Exercise intolerance	◯	◯	◯	◯	◯	◯	◯
Muscle fatigue	◯	◯	◯	◯	◯	◯	◯
Myoglobinuria (dark colored urine)	◯	◯	◯	◯	◯	◯	◯
Rhabdomyolysis	◯	◯	◯	◯	◯	◯	◯
Admission due to McArdle disease	◯	◯	◯	◯	◯	◯	◯

26. While in ketosis do you feel as if you are in permanent 'second wind'?
(Second wind is the condition people with McArdle disease enters after aproximately 6-8 minutes of exercise, where physical activity again becomes easier. Click on the arrow - and select the best answer)

◯ Yes, all the time ◯ Yes, but it comes and goes ◯ Maybe ◯ No ◯ I do not know

27. While in ketosis are your everyday symptoms, related to McArdles disease, improved?
(Click on the arrow - and select the best answer)

◯ I do not know ◯ No improvement ◯ Very small improvement ◯ Moderate improvement
◯ Very high improvement ◯ All my symptoms related to McArdle disease are gone

28. What is the most important effect of the diet?

☐ I have not experienced any effects
☐ Reduction of exercise/activity intolerance
☐ Weight loss
☐ Less muscle pain
☐ Better overall energy level / less fatigue (muscle and/or overall)
☐ Mental clarity
☐ Other
(Click one or more boxes)

If you selected the option "other", please elaborate:

29. If you at any point during the ketogenic diet experienced side effects, please select one or more of the following options:

☐ I have not experienced any side effects
☐ Headache
☐ Nausea
☐ Fatigue
☐ Constipation
☐ Unwanted weight loss
☐ Unwanted weight gain
☐ More menstrual bleeding
☐ Reduced libido (sex drive)
☐ Kidney stone
☐ Gall bladder stone
☐ Frequent urination
☐ Dizziness
☐ Muscle weakness
☐ Other
☐ I do not know
(Click one or more boxes)

If you selected the option "other", please elaborate:

____

30. How are or were the overall side effects?
(Click one or more boxes)

◯ I do not know ◯ No side effects ◯ Small side effects ◯ Moderate side effects ◯ Severe side effects
◯ Very severe side effects

31. Would you advice others with McArdle disease to try a ketogenic diet?
(Click one or more boxes)

◯ I do not know ◯ Yes ◯ Maybe ◯ No

32. How would you rate the overall balance between the efforts versus the effects of the ketogenic diet on your overall functioning?
(Click one or more boxes)

◯ I do not know ◯ The efforts exceed the effects ◯ The efforts and effects degree are similar
◯ The effects exceed the efforts

33. Voluntary: You now have the option to write further information you find relevant related to ketosis or the ketogenic diet:
